# Fabrication, Physical–Chemical and Biological Characterization of Retinol-Loaded Poly(vinyl Alcohol) Electrospun Fiber Mats for Wound Healing Applications

**DOI:** 10.3390/polym15122705

**Published:** 2023-06-16

**Authors:** Camilo Zamora-Ledezma, Ana Belén Hernández, Ivan López-González, Jeevithan Elango, Janèle Paindépice, Frank Alexis, Manuela González-Sánchez, Víctor Morales-Flórez, Duncan John Mowbray, Luis Meseguer-Olmo

**Affiliations:** 1 Green and Innovative Technologies for Food, Environment and Bioengineering Research Group (FEnBeT), Faculty of Pharmacy and Nutrition, UCAM—Universidad Católica de Murcia, Avda, Los Jerónimos 135, Guadalupe de Maciascoque, 30107 Murcia, Spain; 2 Tissue Regeneration and Repair Group, Biomaterials and Tissue Engineering, UCAM—Universidad Católica San Antonio de Murcia, Campus de los Jerónimos 135, Guadalupe, 30107 Murcia, Spain; anabelen136@gmail.com (A.B.H.); ilopez27@ucam.edu (I.L.-G.); lmeseguer@ucam.edu (L.M.-O.); 3 Department of Biomaterials Engineering, Faculty of Health Sciences, UCAM—Universidad Católica San Antonio de Murcia, Campus de los Jerónimos 135, Guadalupe, 30107 Murcia, Spain; jelango@ucam.edu; 4 École Polytechnique Universitaire D’ingénieurs de Montpellier (POLYTECH), Université de Montpellier, Place Eugène Bataillon, 34095 Montpellier, France; janele.p.09@gmail.com; 5 Departmento de Ingenería Química, Colegio de Ciencias y Ingenierias, Universidad San Francisco de Quito (Ecuador), Campus Cumbayá, Diego de Robles s/n, Quito 170901, Ecuador; falexis@usfq.edu.ec; 6 Department of Physics of Condensed Matter, University of Seville (Spain), Av. Reina Mercedes, s/n, 41012 Seville, Spain; mgonzalezs@us.es (M.G.-S.); vmorales@us.es (V.M.-F.); 7 School of Physical Sciences and Nanotechnology, Yachay Tech University, Urcuquí 100119, Ecuador; duncan.mowbray@gmail.com

**Keywords:** adult human MSCs, wound healing, PVA, retinol, drug release

## Abstract

Nowadays, there exists a huge interest in producing innovative, high-performance, biofunctional, and cost-efficient electrospun biomaterials based on the association of biocompatible polymers with bioactive molecules. Such materials are well-known to be promising candidates for three-dimensional biomimetic systems for wound healing applications because they can mimic the native skin microenvironment; however, many open questions such as the interaction mechanism between the skin and the wound dressing material remain unclear. Recently, several biomolecules were intended for use in combination with poly(vinyl alcohol) (PVA) fiber mats to improve their biological response; nevertheless, retinol, an important biomolecule, has not been combined yet with PVA to produce tailored and biofunctional fiber mats. Based on the abovementioned concept, the present work reported the fabrication of retinol-loaded PVA electrospun fiber mats (RPFM) with a variable content of retinol (0 ≤ Ret ≤ 25 wt.%), and their physical–chemical and biological characterization. SEM results showed that fiber mats exhibited diameters distribution ranging from 150 to 225 nm and their mechanical properties were affected with the increasing of retinol concentrations. In addition, fiber mats were able to release up to 87% of the retinol depending on both the time and the initial content of retinol. The cell culture results using primary mesenchymal stem cell cultures proved the biocompatibility of RPFM as confirmed by their effects on cytotoxicity (low level) and proliferation (high rate) in a dose-dependent manner. Moreover, the wound healing assay suggested that the optimal RPFM with retinol content of 6.25 wt.% (RPFM-1) enhanced the cell migratory activity without altering its morphology. Accordingly, it is demonstrated that the fabricated RPFM with retinol content below the threshold 0 ≤ Ret ≤ 6.25 wt.% would be an appropriate system for skin regenerative application.

## 1. Introduction

The skin is the largest organ in most vertebrate organisms, having a complex structure that under normal conditions exhibits the capacity of self-regeneration. This organ is involved in multiple biological processes; for example, it regulates body temperature, supports blood vessels and nerves, prevents dehydration, detects sensory signals, and acts as an external barrier against pathogens and chemicals [1]. Due to its extensive involvement, the skin always tends to become damaged (wound) by several external and internal factors. By definition, a wound is a disruption of the normal anatomical and physiological structure of tissue and represents considerable damage to the natural barriers of defense against foreign agents [1]. Wound healing is a specific complex biological process related to the general phenomenon of tissue growth and regeneration. It is a priority and a challenge for specific individuals with chronic diseases such as diabetes, immunosuppression, collagenopathies, vasculopathies, malnutrition, radiotherapy, and aging, or for those individuals with a natural tendency to suffer from wounds that are difficult to heal or those who produce pathological scars (keloids) [2,3,4,5]. Thus far, many routes have been used as treatments including but not limited to the use of specific medical instruments, cell therapies, tissue transplantation, and wound dressings [4,6]. These approaches exhibit advantages and disadvantages, making it difficult to reach a consensus on the most appropriate treatment to achieve wound healing. The main drawback that must be faced with the aforementioned therapies lies in the fact that most of them are expensive and often require sophisticated infrastructures and highly specialized professionals [6,7]. On the contrary, wound healing treatments based on the use of biomaterials such as hydrogels, pads, or mats remain as some of the most readily available and affordable therapies [2,5,8,9,10,11,12]. Thus, the development of cost-efficient biomaterials based on the association of polymers with bioactive molecules (BAM) aiming to both protect a wound and also improve the healing process remains one of the most exciting research topics in biomedical sciences [3,4]. In general, an ideal wound healing dressing should possess the following properties: (1) absorb exudates and toxic components of superficial wounds; (2) preserve a controlled environment with a high level of moisture necessary at the wound surface; (3) allow gas exchange; (4) provide thermal insulation; (5) protect the wound from secondary bacterial contamination (secondary infection); (6) be non-toxic; (7) promote regeneration and growth of new tissue; and (8) be easily removed without causing secondary trauma to the wound [13,14,15,16]. Nowadays, it is even more appealing to produce wound dressing materials aiming to improve the management of the healing [2,4,6,7,10]. Moreover, the increasing sustainability, environmental and cost-benefits concerns increase the demand for the fabrication of innovative materials. Thus, it is expected that the designed biomaterial would positively impact any of the four main phases of the healing process: (i)—hemostasis, the phase at which the wound is closed by platelet-mediated coagulation phenomenon. This process starts when blood leaks out of the body when blood vessels constrict to restrict the blood flow; (ii)—inflammation, characterized by macrophage/leucocytes neutrophils/lymphocytes infiltration and immune system activation accompanied by cytokine production and growth factors, (iii)—proliferation, characterized by the removal of the non-viable damaged tissue (necrotic), increases in angiogenic activity, extracellular matrix synthesis, and the formation of granulation tissue beginning at the wound site, and (iv)—remodeling, characterized by the formation and contraction of scar tissue which is the main indicator of the completion of the wound healing process [2,3,17,18].

Presently, there is a trend to develop cell-free skin substitutes which fulfill the functions of the extracellular matrix, cytokines, and growth factors, in addition to providing protection and support during wound healing. In this regard, many types of wound dressing have been developed thus far ranging from gels, hydrogels, hydrocolloids, films, membranes, nano/microfibers, and fiber mats fabricated either from the association of natural/synthetic polymers with natural/synthetic bioactive molecules or nanomaterials [3,6,8,19,20,21,22]. These bio-friendly materials having similar properties as skin can be engineered as compresses or adhesive dressings, which are simply applied to the skin [2,3]. Likewise, different materials were developed to protect a superficial wound by using a dressing in the form of a spray that produces a plastic film on the skin for several hours [3,4]. In a similar approach, another appealing alternative consists of combining the abovementioned biomaterials with either cells or the fabrication of cell sheets that secrete extracellular matrix (ECM), which facilitates the growth and proliferation of cells [2,17].

These biomaterials can be formulated through different routes, including but not limited to the fabrication of nano/microfiber or fiber mats by electrospinning, which deserves special attention in wound dressing applications because it allows fabricating industrially scalable porous 3D structures that can mimic the structure of the skin extracellular matrix (ECM). The latter architecture is highly compatible with the adsorption of wound exudate, nutrient, and gaseous exchanges that would prevent bacteria invasion/proliferation at the wound site. Indeed, electrospinning technique allows the transformation of polymer solutions into tailored nano/micro-sized fiber mats by applying a high voltage electric field [23]. In addition, such fiber mats can be functionalized with bioactive molecules to enhance their wound healing capacity depending on the intended use [7,24,25,26,27]. Moreover, electrospinning technology has advanced enormously over the last decade; thus, at the present date, it is a very versatile technique that not only is used to produce single fiber mats, but can also be used to produce coaxial fiber (core-shell) architectures, as well as multijet to produce a fiber mats made of entangled fibers with different formulations and porosities, among others [23,28,29].

A typical set-up for electrospinning consists of a syringe with a needle as an electrode, connected to a voltage source and a collecting surface. Once the applied voltage is greater than the solution’s surface charge, it will be projected towards the collecting surface. Optimal conditions occur when a single Taylor cone is formed. Actually, during this process, the solvent in the solution evaporates, the polymer solidifies and can be collected on the opposite electrode, in the form of fiber, bead, or a combination of these structures, depending on different factors related to (i)—experimental set-up, (ii)—environmental conditions, (iii)—solution’s physico–chemical properties [30,31,32]. As a matter of fact, many polymers both from synthetic/natural sources have been tried in the fabrication of electrospun-based wound dressing materials such as silk fibroin, keratin, chitosan, collagen, hyaluronic acid, casein, poly(lactic acid) (PLA), poly(lactic Acid) (PGA), poly(lactic-co-glycolic acid) (PLGA), poly(methoxydiethyleneglycol methacrylate) (PDEGMA), polyvinylpyrrolidone (PVP), polycaprolactone (PCL) [33], and polyvinyl alcohol (PVA), among others [24,25,34]. Likewise, to enhance the healing performance, their combination or functionalization with different synthetic/natural bioactive molecules have also been tried, including but not limited to vitamins, growth factors (GFs), antibiotics (Ciprofloxacin (CIF), gentamycin, tetracycline hydrochloride (TCH), silver sulfadiazine (SSD), nanomaterials such as silver nanoparticles, and natural extracts such as thymol, cinnamon, clove, lavender, rosemary, oregano, lemongrass, peppermint, tea tree, and manuka [23,24,35].

Among the aforementioned polymers and bioactive molecules, PVA stands as a very good polymer precursor for electrospinning because it is water-soluble and biodegradable with a molecular weight ranging from 20,000 to 200,000 D and is used frequently in biomedical applications. As reported in the literature, PVA incubated in PBS degrades up to 40% in 30 days [36,37]. However, its degradation time or biodegradation are dependent on many factors such as the degree of hydrolysis, temperature, medium, and microorganism used during experiments [38]. PVA can be produced following the polymerization of vinyl acetate or following a radical polymerization of vinyl formate, vinyl pivalate, and vinyl trifluoroacetate. PVA is a semi-crystalline polymer with a glass transition temperature of 85 °C and a melting temperature of 200 °C [19,39,40,41]. Recently, Serbezeanu et al. reported the fabrication of electrospun fibers combining PVA with *Thymus vulgaris*, *Salvia officinalis folium*, and *Hyperici herba* aiming to produce fiber mats with phytotherapeutic agents. Authors claimed that such materials were readily fabricated, and they could be designed in the form of a variety of sizes and shapes, being entirely foldable, with potential antibacterial and antifungal capacity against *S. aureus*, *E. coli*, and *Methicillin-resistant staphylococcus aureus* (MRSA) [42]. Similarly, Sarwar et al. reported the advantages of a novel electrospun fiber membrane, with enhanced antibacterial and cytocompatibility properties employing PVA, copper II oxide nanoparticles (CuONPs), and *Momordica charantia* (bitter gourd) extract [43]. Parın et al. also reported the formulation of biocompatible PVA electrospun membranes by the association with gelatin, polyvinyl pyrrolidone (PVP), and different amounts of folic acid (FA), and claimed that the utilization of hydrophilic fibers would impact significantly the vitamin’s release, making them a promising material which can be utilized as fast-dissolving transdermal delivery systems [44].

Likewise, among the different bioactive molecules employed thus far, the use of retinol (vitamin A) stands as an interesting biomolecule for wound dressing applications due to its concomitant physico–chemical and associated biological compatibility. Indeed, retinol is an isoprenoid similar to vitamins E, K, and cholesterol, all of which are formed from isoprene units, and considered as hydrophobic but fat-soluble. Actually, retinol is a hydrocarbon molecule with a single hydroxyl group at one end, which can be oxidized to form an aldehyde group or to form a carboxylic acid group to produce retinoic acid, which is the biologically active form of retinol [45,46,47,48]. This vitamin is widely used in the field of cosmetics and tissue engineering. For instance, retinol has been used in many beauty products such as creams due to its anti-aging and defensive properties [47,48,49]. Thus, retinol plays an important role in the regeneration of the skin by regulating the inflammatory response of the human body, the reparative collagen synthesis in the incisional wound, and angiogenesis. It has been demonstrated that retinol stimulates the renewal of the epidermis but also increases the rate of re-epithelialization in wounded skin and thus restores the epithelial structure. Whether ingested or applied topically, the action and effectiveness of retinol remain the same [50].

In this context, Li et al. reported the manufacture of highly biocompatible electrospun fiber mats based on the association of gelatin with vitamins A and E. They claimed that such materials were suitable as wound dressing based on their improved cell adhesion and proliferation in the early stages of culture [51]. Similarly, Müller et al. demonstrated that electrospun mats based on poly(D, L-lactide) (PLA) loaded with Ca-polyphosphate/retinol nanospheres exhibited huge potential as biomaterial due to their enhanced morphogenetic activity, along with the antibacterial properties [52]. All the above reports disclosed that nowadays there exists a huge interest in producing innovative, high-performance, biofunctional, and cost-efficient electrospun biomaterials for wound dressing applications. In fact, the actual interaction mechanism between the skin and the wound dressing material remains unclear. However, most researchers and industries agreed about the benefits of producing tailored electrospun mats because of their breathable nature, flexibility, better mechanical properties, and biological response, which would play a key role in the wound healing process.

Based on the abovementioned concept, the present work is focused on the fabrication of retinol-loaded poly(vinyl alcohol) (PVA) electrospun fiber mats (RPFM) with a variable dry retinol content (0 ≤ Ret ≤ 25 wt.%). Furthermore, the present study describes the synthesis, physical–chemical and biological characterization of fabricated RPFM through electron microscopies, thermogravimetric analyses, and tensile tests, followed by in vitro cumulative release experiments, cytotoxicity, and biocompatibility using primary culture of adult human bone marrow-derived multipotent mesenchymal stem cells (*ah*-BM-MSCs).

## 2. Materials and Methods

### 2.1. Materials

Fully hydrolyzed PVA, molecular formula [-CH_2_CH(OH)-]_n_, (molecular weight approximately 60,000), CAS number: 9002-89-5, stock keeping unit (SKU): 8438661000 was acquired from Sigma Aldrich (St. Louis, MO, USA). Hydrolyzed retinol (molecular formula C_20_H_30_O, CAS:68-26-8) was acquired from NA Beautiful Store, Shanghai, China.

### 2.2. Retinol-Loaded Poly(vinyl Alcohol) Fiber Mats (RPFM) Fabrication

Fiber mats were fabricated from a set of polymeric aqueous solutions of PVA loaded at different contents of retinol. All the solutions were fabricated using an aqueous PVA solution at 15 wt.%. The specific concentration of PVA was chosen based on the earlier literature [53] in order to obtain single fiber morphologies. In brief, 15 wt.% PVA solution was prepared by mixing 30 g PVA with 170 g distilled water (on weight basis) and heated in an oven overnight at 90 °C to obtain complete solubilization. PVA solutions with different retinol contents were obtained by adding the desired amount of hydrolyzed retinol powder directly to 15 wt.% PVA solution and homogenized in a magnetic stirrer at room temperature for 15 min. A total of five types of aqueous mixtures varying in retinol content in the range of 0.0 wt.% ≤ Retinol ≤ 5.0 wt.% were fabricated. Table 1 summarizes the sample weight% of PVA and retinol in the aqueous mixtures.

The aforementioned aqueous PVA/retinol mixtures were employed to fabricate the set of retinol-loaded PVA fiber mats (RPFM). Typically, 10 mL of the aqueous mixtures were introduced into a 24 mL syringe adapted to the electrospinning machine. A detailed description of the electrospinning set-up employed is described previously [29,30,32,54]. In the present work, the parameters of the experimental set-up were chosen to obtain a stable Taylor cone. In a typical procedure, the PVA retinol-loaded membranes were obtained by using the electrospinning horizontal set-up following the operational parameters: (i)—tip-to-collector distance equal to 18.2 cm, (ii)—syringe diameter of (20.05 mL), (iii)—target volume ranging from 1 to 3 mL depending on the final intended thicknesses; (iv)—flow rate equal to 0.38 mL/h, and (v)—applied voltage equal to 19 kV. In addition, the same operational parameter conditions were followed for all five different aqueous mixtures varying in retinol content. The collecting surface was also covered with aluminum foil to guarantee that membranes were readily peeled off. Fiber mats fabricated in about 3 h exhibited 100 to 300 µm of thickness.

### 2.3. Characterization

#### 2.3.1. RPFM Chemical Composition and Thermal Stability

The chemical composition and thermal stability were investigated by TGA-SDT analysis (TA instruments, New Castle, DE, USA, SDT Q600), operated at a maximum temperature 900 °C, 10 °C/min, under N_2_ atmosphere.

#### 2.3.2. RPFM Morphology

The microstructure of the fiber mats was captured by Scanning Electron Microscopy (SEM) with a FEI (Thermo Fisher Scientific, Waltham, MA, USA)Teneo device, working with 2 kV of acceleration voltage. Samples were Pt-coated for an adequate inspection. Diameters of the fibers were estimated with the help of ImageJ software(The National Institute of Health, New York, USA), measuring more than 300 fiber diameters among 5 to 6 different images of each sample. At least 3 to 5 different Regions of Interest (ROI) were examined for each sample in order to corroborate the homogeneity. Uncertainties in the fiber diameter are considered as standard deviation.

#### 2.3.3. RPFM Mechanical Properties

Mechanical properties of the RPFM were obtained through tensile tests using a Texture Analyzer (Brookfield Ametek, Model: CT3-50KG, Middleboro, MA, USA) with a load cell range of 50 kg equipped with the TA-RCA Roller Cam accessory grips, which allow the pulling apart of a sample using tensile force to measure the tensile strength and tear characteristics of a given material including thin polymer films. The operational parameters of the experiment were: 70 mm as the target distance for the grips, 10 s as hold time, 0.1 N trigger force, and 0.1 mm/s test speed. Tensile tests were performed at least in triplicate for all the samples, with typical specimen dimensions of 100–300 µm height (thickness), 8 mm width, and 70 mm length. The following mechanical parameters were obtained from the stress–strain curves: Young’s Modulus (*E*) as the slope of the initial linear regime, which provides information about how much stiffer the material is in the elastic zone; the Ultimate Tensile Strength (UTS), which measures the maximum load that a material can bear before necking or breaking, and the elongation at break (strain, ε*_max_*), also known as fracture strain, which provides information regarding the ratio between the initial/final length after breakage of the sample, or the capability of the material to resist changes of shape without crack formation.

### 2.4. Retinol Release Quantification

To compute the amount of retinol released over 1 to 7 days for retinol-loaded PVA fiber mats, RPFM-0.1, RPFM-0.5, RPFM-1, and RPFM-5 to the medium, a typical calibration curve for the retinol in solution in the range of 5 mg/L < X < 200 mg/L concentration was established. Then, its curve equation from a linear regression given by *Y = m X + b*, with *R*^2^ > 0.99, was obtained by using UV-Vis spectroscopy. In the latter equation, *X* represents the retinol concentration (mg/L), *Y* is the corresponding UV absorbance intensity measured, and the constants of *m* and *b* are the slope and intercept of the curve calculated from its linear regression, respectively. UV-vis spectra were recorded using a UV-1800 spectrophotometer (Shimadzu Corporation, Tokyo, Japan). Quartz cells with an optical path of *d* = 10 mm were used in all the experiments. Spectra were recorded within the wavelength range of 215 nm to 800 nm and systematically background corrected. All the in vitro assays were carried out using phosphate-buffered saline solution (PBS-Gibco (Thermo Fisher Scientific, Waltham, MA, USA) [18,55]. Fiber mats loaded with different contents of retinol were cut into pieces of approximately 2.5 cm^2^ and weighted (around 60 mg for each sample researched) following typical recipes as previously reported [18,56,57]. The release rate of retinol was investigated for 7 days following the typical recipe for similar in vitro assays [3,18,57]. In a classic procedure, the fiber mats pieces were incubated in 10 mL of a PBS solution with pH~7.4. Samples were kept at 37 °C and at different time points, 2 mL of the solution was withdrawn for UV-vis analysis and replaced by the same volume of 2 mL of fresh PBS solution. Subsequently, the UV intensity absorption at 335 nm was recorded. The accumulative release was calculated using the following formula:

(1) $$CR\left(\mathrm{mg}\right)=10\left(\frac{Y\;-b}{m}\right)+2\sum_{i=1}^{n}R_{i-1}$$

in which *CR* represents the cumulative amount of retinol released to the medium in mg, the “*Y* ” represents the absorbance intensity measured, *m* and *b* are the slope and intercept of the curve calculated from its linear regression, and *R_i_* represents the amount of retinol in mg that was removed (discarded) from each previous time interval sampling and replaced with fresh PBS. It is important to stress that the units for the retinol concentration obtained through UV-vis spectroscopy must be in mg/mL. The subscript *i* refers to the specific period of time researched. The prefactor terms appearing as 10 and 2 refer to the volume used for release experiments, and the volume discarded at each period of time, respectively. Finally, the cumulative release percentage *M*(*t*) was obtained by using *M*(*t*) = *CR/C*_0_ × 100 in which C_0_ represents the initial retinol content in the sample investigated.

### 2.5. Cellular Assays

All the in vitro cell culture was followed as per the regulatory guidelines of the Institutional Ethics Committee of UCAM-Universidad Católica de Murcia UCAM and approved on 28. May 2022. (CE No 052114/05.28.2022). Cells were kindly donated by the cell therapy group of the IMIB-Hospital Clínico Universitario Virgen de la Arrixaca (Murcia), and all the surgical procedures of bone marrow collection, cell isolation, and phenotype characterization were reported in our previous work. Informed consent was obtained from all subjects involved in the study. Then, cells in passages 3 and 4 (P3–P4) were incubated at 37 °C in a 5% CO_2_ atmosphere and 95% of relative humidity and characterized in accordance with the ISCT guidelines [20,58,59] to be used in all subsequent in vitro assays. The morphology of the live cells was observed with an inverted microscope. It is worth noting that multipotent adult stem cells have been used because they exhibit proliferative potential, produce proteins, cytokines, and growth factors, but also in addition, they can differentiate into various cell types including those involved in the wound healing process [60,61]. Prior to in vitro studies, all the test samples were sterilized under UV for 15 min by using a UV Sterilizer, 9 W, 60 Hz (SKU: OT-HSYXF-2624-EU, model YM-9002, Shanghai, China). For viability, proliferation, and scratch assays, *ah*-BM-MSCs were seeded at the bottom of 24-WP tissue culture-treated polystyrene wells (TCPS; Sigma-Aldrich, Corning, NY, USA), and the RPFM were placed in 0.4 μm pore culture well inserts (Falcon^®^, Corning, New York, NY, USA) to be in indirect contact with cells. Cells seeded onto wells without RPFM treatments were considered a positive control. The media were changed twice a week for all the experiments performed.

#### 2.5.1. In Vitro Cytotoxicity Assay

The viability of *ah*-BM-MSCs was evaluated using an LDH cytotoxicity detection kit (#11644793001, Roche Diagnostics, Roche. Applied Science, Mannheim, Germany), according to the manufacturer’s instructions. Before performing the assay, the optimal cell concentration was determined by seeding *ah*-BM-MSCs at different densities (ranging from 100 to 1 × 10^5^ cells/well) on a 24-well plate. The optimal seeding concentration of *ah*-BM-MSCs was at a density of 3 × 10^4^ cells/well. Briefly, 24 h after seeding (to allow cell adhesion), the assay medium containing 1% FBS was replaced by fresh assay medium in order to remove LDH activity released from the cells during the incubation step. Then, the RPFM were placed in culture well inserts (0.4 μm pore) to facilitate the retinol release into culture medium for cell growth, and then LDH activity was assessed at 1, 24, 48, and 72 h. Cells culturing onto TCPS (without RPFM) were used as low control (spontaneous LDH release), and cells treated with Triton X-100 solution (2% in assay medium) were used as high control (maximum LDH release). At different time points, 100 μL aliquots of each well were transferred to an optically clear flat-bottomed 96-well plate followed by the addition of 100 μL of the reaction mixture. After 30 min incubation at RT, the absorbance was read directly in a Spectramax iD3 plate reader (Molecular Devices, San Jose, CA, USA) at 490 nm.

#### 2.5.2. In Vitro Cell Proliferation

The cellular metabolic activity of *ah*-BM-MSCs cultured in presence of RPFM was evaluated using the AlamarBlue^®^ assay (#DAL1100, Invitrogen, Carlsbad, CA, USA) on days 1, 3, 7, and 14 after seeding. Cells were cultured on a 24-well plate at a density of 3 × 10^4^ cells/well. Cells onto TCPS (without RPFM) were used as control. Briefly, at different study periods, the inserts containing RPFM were temporarily removed (prior to the incubation step), and fresh medium (1 mL) containing 10% (*v*/*v*) AlamarBlue^®^ reagent was added to each well. Then, the plate was wrapped with aluminum foil (to provide a dark environment) and incubated at 37 °C in a 5% CO_2_ atmosphere with 95% of relative humidity for 4 h. After the incubation step, 150 μL aliquots of each well were transferred to a black-walled 96-well plate, and fluorescence was measured in a Spectramax iD3 plate reader (Molecular Devices, San Jose, USA) at excitation and emission wavelengths of 530 and 590 nm, respectively.

#### 2.5.3. Wound Healing Assays

The scratch assay is the most common in vitro method for testing molecules with anti- and pro-cell migration properties due to its polyvalence, cost-effective method, and ease of performance on adherent cell lines, such as *ah*-BM-MSCs. In the present work, the migration of *ah*-BM-MSCs was assessed by an in vitro scratch wound closure assay in which a scratch was generated, on a two-dimensional confluent cell monolayer, at different study periods (0, 24, and 72 h). Cells were cultured on a 24-well plate at a density of 1 × 10^4^ cells/well and incubated at 37 °C and 5% CO_2_ for one week to permit cell adhesion and the formation of a confluent cell monolayer (90–95%). Then, the medium was removed, and a longitudinal wound was made with the help of a ruler and a 200 µm sterile pipette tip generating a gap of approximately 500 ± 50 µm in width. Subsequently, the inserts containing the RPFM loaded with different retinol concentrations were placed in each well, and the plates were incubated after addition of fresh culture medium. At each time period, wound closure was verified by capturing images using an inverted optical microscope coupled with a digital camera, Axiocam 305 mono (Axio Vert A1, Serial No 3847016567, Carl Zeiss Microscopy GmbH, Suzhou, China).

**Figure 1:**
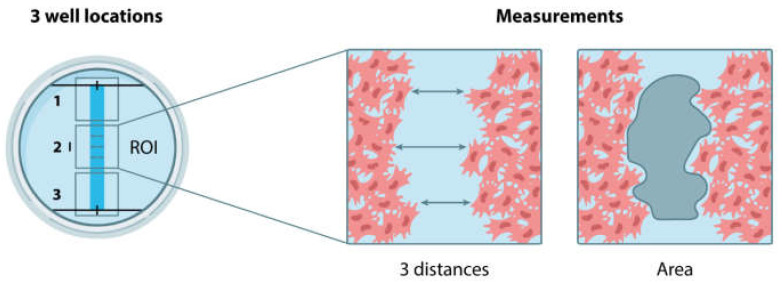
Schematic representation of the scratch wound healing assay performed. The well locations 1 and 3 were discarded as they are considered as artefactual areas (not homogeneous areas). The well location 2 was taken as the region of interest (ROI). All the scratch assays were performed in triplicate.

Wound closure was monitored by collecting images at the different study periods (0, 24, and 72 h) after the scratch was performed. Then, wells were rinsed three times with PBS and wound closure was calculated by two ways: (i) determining the distances between edges at reasonable intervals (*n* = 3), and (ii) measuring the gap area bounded by the wound edges. For the first case, the initial measure was quantified using the black mark made on the bottom of the well as a reference, and the two successive measurements were made at a distance of 400 ± 50 microns above and below the mark in Figure 1.

For its part, the migration rate can be expressed as the change in the wound area over time or as the percentage of area reduction over time (wound Closure% Equation (2)). The wound area can be readily computed by ImageJ software as follows:

(2) $$Wound\;Closure\left(\%\right)=\left[\frac{A_{t=0h}-A_{t=∆h}}{A_{t=0}}\right]\;\times \;100$$

where *A_t=0h_* denotes the area of the wound measured after scratching (*t* = 0 h) and *A_t=_*_Δ*h*_ is the area of the wound measured Δ*h* hours after the scratch is performed [62,63].

### 2.6. Statistical Analysis

All data presented through this work were analyzed using GraphPrism 9.0.1 (GraphPad Software Inc., San Diego, CA, USA). The statistical significance was determined by a two-way ANOVA, and comparison between groups was evaluated with *t*-tests (*p* < 0.05). Experiments were carried out at least in triplicate (n = 3) unless otherwise specified, and all results were expressed as a mean ± standard deviation.

## 3. Results and Discussions

### 3.1. Fabrication and Optimization of Retinol-Loaded Poly(vinyl Alcohol) Fiber Mats (RPFM)

**Figure 2:**
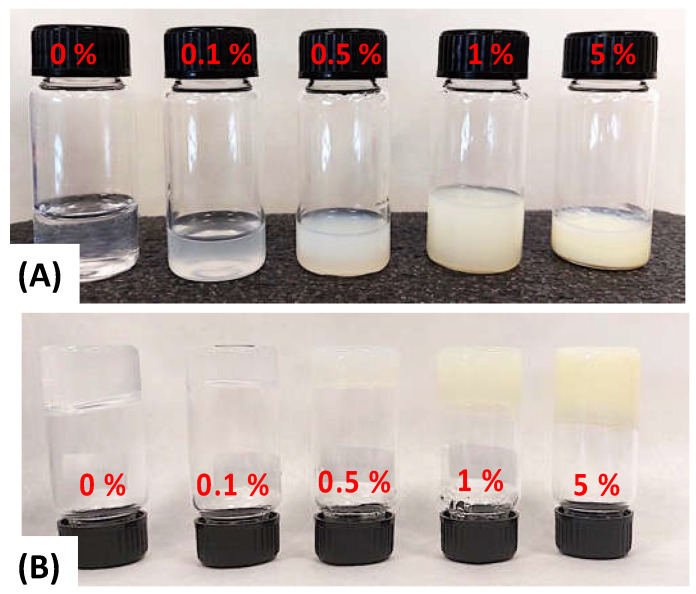
Typical macroscopic picture of vials containing ~10 g of five different aqueous mixtures varying in retinol content in the range of 0.0 wt.% ≤ Retinol ≤ 5.0 wt.% at different temperatures (**A**) room temperature, and (**B**) at 5 °C. All samples contain a fixed amount of 15 wt.% of PVA. Samples at 5 °C exhibited gel-like structure.

In this study, hydrolyzed retinol was specifically chosen due to its cheaper cost and higher solubility in aqueous solution to provide a novel route to fabricating readily available, reproducible, and affordable biomaterial membranes with potential application in the clinical area [29,62,63,64,65]. Table 1 summarizes the weight% (wt.%) of PVA and retinol present in the aqueous mixtures and their corresponding retinol-loaded PVA electrospun fiber mats (RPFM). Likewise, Figure 2 displays a typical macroscopic picture of vials containing ~10 g of the aqueous precursor mixtures employed. As observed, the sample without retinol was completely clear and transparent (Figure 2a). In contrast, as the retinol content increased, the sample became a little bit opaque/blurry until it turned into a white color for the aqueous mixture containing retinol above 0.5 wt.%.

It is worth mentioning that samples were freshly prepared prior to use, in order to avoid undesired retinol degradation or phase separations, because it is well known that the retinol tends to degrade easily by light, temperature, and pH exposure [46,47,49,50]. We also noted that all these samples exhibited interesting rheological properties. At room temperature and above, i.e., 37 °C, samples flow similar to a thick solution. In contrast, the samples exhibited a typical physico-gel-like or hydrogel structure at 5 °C (Figure 2b), which is also highly appealing for tissue engineering applications [8]. Indeed, it has been demonstrated that similar PVA hydrogels would be suitable for the development of novel biomaterials with antimicrobial properties and also for the adhesion and proliferation of mesenchymal stem cells (MSCs) [8].

### 3.2. RPFM Scanning Electron Microscopy (SEM)

**Figure 3:**
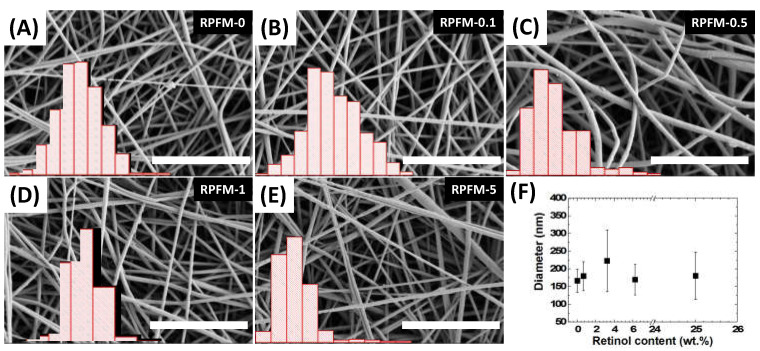
(**A**–**E**) Selected scanning electron microscopy images for the retinol-loaded PVA fiber mats. Scale bar corresponds to 5 μm in all cases. Insets show histograms of the diameters of the fibers, spanning over 40–300 nm for samples RPFM-0 and RPFM-0.1; 50–600 nm for RPFM-0.5; 0–350 nm for RPFM-1; and 50–550 nm for RPFM-5, respectively. (**F**) Average fiber diameter for each sample, as a function of the content of retinol. Standard deviation was considered as well as the uncertainties.

All the fiber mats were fabricated from the abovementioned set of polymeric aqueous mixtures of PVA loaded with different contents of retinol [29,30]. The inspection of the fibers through scanning electron microscopy allowed the analysis of the fiber diameters as well as checking their homogeneity at the microscale. Figure 3 shows the representative images of the retinol-loaded PVA fiber mats (RPFM) and their superposed fiber diameter distributions. Through SEM observations, the existence of single fiber morphologies without the presence of beads in the sample investigated was confirmed. According to the literature, such bead-like defects within their microstructure can often alter their concomitant physico–chemical properties including but not limited to the mechanical properties and their biological activity [24,63,64,65]. Furthermore, it is worth mentioning that aqueous polymeric precursor mixtures exhibited a conductivity around 60 mV for native PVA aqueous solution at 15 wt.%, while the addition of retinol resulted in increases up to 90 mV. However, such variations did not affect the fiber morphologies [66].

In addition, it was demonstrated that on average, the diameters’ distribution of the fibers did not exhibit significant changes: i.e., diameter distribution ranged from 150 nm up to 225 nm in all cases. Interestingly, the increase in the dispersion of fiber diameters’ distribution was superior for samples RPFM-0.5 and RPFM-5, in which some fibers’ diameters were thicker than 400 nm, suggesting a bimodal diameter distribution. In contrast, the RPFM-1 sample did not exhibit this characteristic, which impeded us from ascribing this feature in terms of the presence of retinol in the samples. Such morphologies and diameters are in total agreement with those expected from literature. For instance, Ince-Yardimci et al. recently fabricated PVA electrospun fiber blends with single fiber morphology and diameters’ distribution ranging from 150 to 200 nm [67].

### 3.3. RPFM Thermogravimetric Analyses (TGA)

**Figure 4:**
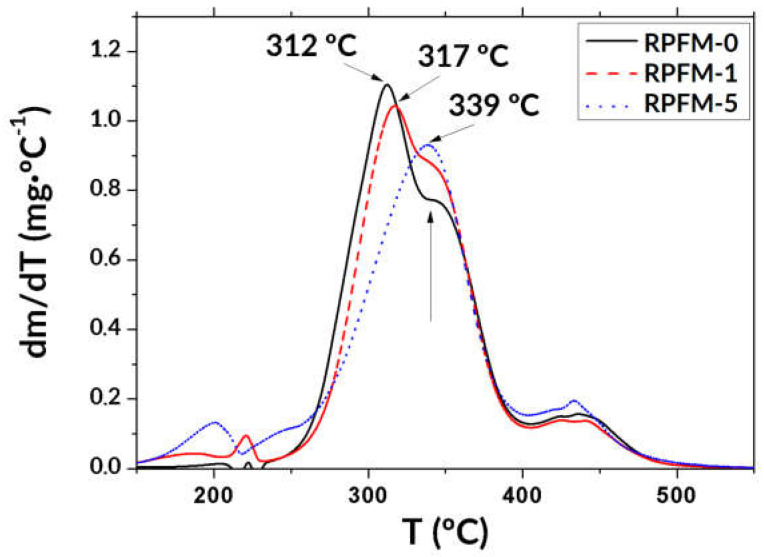
Derivatives of the curves from thermogravimetric analyses of three selected samples (RPFM–0, RPFM–1, and RPFM–5). Vertical arrow points to the disappearing second degradation step of the pure PVA samples.

Thermogravimetric analyses revealed the thermal stability of the polymeric fiber materials and the effect of the retinol. The derivative of the mass variation versus temperature curves corresponding to three selected samples (RPFM-0, RPFM-1, and RPFM-5) is shown in Figure 4.

As observed, the preliminary weight loss occurred between 25 and 250 °C in all tested samples. Such features are attributed to the loss of water present in the form of humidity or moisture. In addition, samples exhibited good stability up to 150 °C, but above this temperature, the retinol started to slightly affect the corresponding thermogram. First, a small loss of mass between 150 °C and 230 °C appeared in retinol-containing samples, about −4% for sample RPFM-1 and −6% for sample RPFM-5. The total degradation of the samples was found between 230 °C and 500 °C, typically attributed to the cleavage of backbone polymeric chains. Moreover, the temperatures at the maximum mass loss rate were observed at 312, 317, and 339 °C for the samples RPFM-0, RPFM-1, and RPFM-5, respectively, indicating that the thermal stability as well as the degradation temperature of the samples were affected by the presence of retinol. The aforementioned temperature trend is often associated with the fiber crystallinity, suggesting that the present retinol-loaded fiber mats are more effective in promoting some crystallization, but also demonstrate the presence of some intermolecular interactions between the PVA chains and the crystalline structure of the samples [68,69]. Furthermore, these thermal stability changes reveal that the side chains of PVA are more difficult to decompose prior to the main chains in the samples. In addition, the RPFM-0 sample (PVA native) exhibited a two-step thermal degradation process in this range of temperatures. It is worth noting that the second step progressively disappeared (vertical arrow in Figure 4), probably masked due to the shifting rightwards of the curve as the retinol content increased. This result clearly indicates that this peak is due to the loss of water from the network, which is usually associated with the water trapped within the crystalline structure due to the presence of guest molecules embedded in the PVA matrix, which is consistent with the previously reported works [68,70].

Lastly, a final degradation step appeared at 440 °C in all cases. These results suggested that RPFM exhibited an improved thermal stability compared to their native PVA, which was in agreement with the previous report [71]. To support the present study, Rajora and Bal [72] found a similar weight loss of ~61% between 25 and 200 °C for PVA electrospun fibers and further reported that the PVA/cashew gum polysaccharide (CGP) fibers had a highest degradation (~48%) at 350 °C and complete degradation at 550 °C, demonstrating the improved thermal stability of PVA/CGP fibers in comparison to the native PVA fibers [72].

### 3.4. RPFM Mechanical Properties

**Figure 5:**
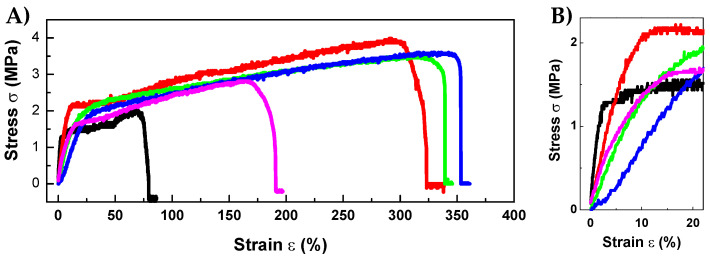
(**A**) Representative stress σ versus strain ε plots of tensile tests for retinol-loaded PVA fiber mats varying in retinol content: RPFM-0 (black), RPFM-0.1 (red), RPFM-0.5 (green), RPFM-1 (blue), and RPFM-5 (magenta). (**B**) Shows a magnification of the data in the 0–30% of strain region for clarity.

An ideal biomaterial for wound healing applications must adhere well to the surrounding tissues depending on their final intended use. Therefore, their mechanical properties should play a crucial role and must be close to that of the native tissue to prevent any undesired damage, breakage, or detachment during the whole healing process. Thus, it is crucial to assess the most important mechanical properties of the manufactured biomaterials [19]. Figure 5 shows the stress σ in MPa versus strain ε in % plots of the tensile test of the samples. Likewise, Table 2 shows the summary of the mechanical properties of the RPFM with different contents of retinol. Surprisingly, the Young’s modulus decreased significantly with increasing the content of retinol (i.e., Young’s modulus values of RPFM-0 (44.73), and RPFM-1 (8.13), or RPFM-5 (9.20)), indicating that the material becomes less stiff within the elastic regime, i.e., a decrease in the elastic modulus of the material. Based on these results and the suggested improvement of the crystalline regions in the composites induced by the presence of retinol as described in the preceding section, it is evident that in the present case, different mechanisms behind the lower stiffness exhibited by retinol-loaded nanofibers exist. We hypothesize that the present trend would involve a combination of intermolecular interactions of the PVA chains with the crystalline regions, but also, the presence of retinol can cause an alteration in the distribution of amorphous and crystalline regions [68,69,73]. Nevertheless, this trend was also reported in similar materials in which polymeric fiber mats were loaded with different molecules or nanoparticles. Previously, it has been reported that such behavior is ascribed to the partial modification of the polymeric structure due to the presence of the guest molecule [74,75]. In support of this study, Huang et al. reported previously that the elastic modulus of citric acid crosslinked electrospun PVA membranes were systematically slightly lower than that of the native PVA [76]. On the contrary, the ultimate tensile strength of the samples increased up to 170% for the RPFM-1 compared to their native PVA fiber mats. These results suggested that the cross-linking between retinol and PVA molecules is mainly through hydrogen bonds, providing a denser structure within the fibers, provoking a sample that can resist a larger load before necking [39]. Conversely, the elongation at break was enhanced by about 380% for the RPFM-1 samples, i.e., an increase of about fivefold compared to native PVA fibers. Nevertheless, the elongation at break of the samples with higher retinol content (RPFM-5) decreased, demonstrating the dependence of the mechanical properties due to the presence of retinol. To support the present results, many studies reported similar elongation at break trends for analogous systems containing different kinds of molecules, bioactive molecules, nanoparticles, or nanocarbonaceous structures as guest additives [29,32,77,78]. In this context, Goudarzi et al. recently found the increase of the elongation at break of kappa-carrageenan-PVA electrospun fiber mats loaded with prunus domestica anthocyanins and/or epigallocatechin gallate compared to their non-loaded PVA electrospun fiber mats [79]. Therefore, the elongation at break of fiber mats depends on the nature of the biomolecules loaded into the fiber mats. All these results suggested that the mechanical properties of these mats can be tailored by tuning the retinol content, in order to match the mechanical properties of the target tissue. Moreover, it is demonstrated that those RPFM containing retinol below or equal to 6.25 wt.% exhibited significant improvement in their mechanical properties, which can be associated with the fact that as retinol content increases, the PVA content slightly decreases [23], lowering the concentration of PVA in the precursor solution and therefore minimizing its elastic resistance [66,80].

### 3.5. Retinol Release Quantification

First, a retinol calibration curve following Beer–Lambert’s law was performed, allowing the determination of the retinol content in samples with unknown concentrations by simply measuring its absorbance intensity.

**Figure 6:**
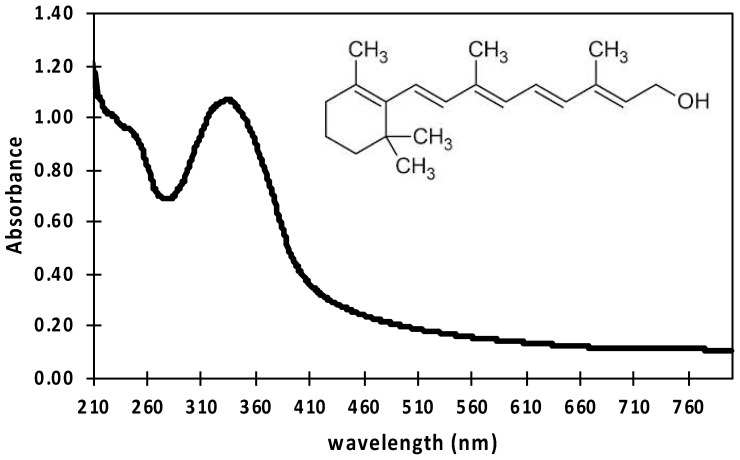
Typical UV-Vis absorbance spectrum of an aqueous retinol solution in PBS at 200 mg/L with the major absorption peak located at 335 nm. The inset shows the chemical structure of retinol.

**Figure 7:**
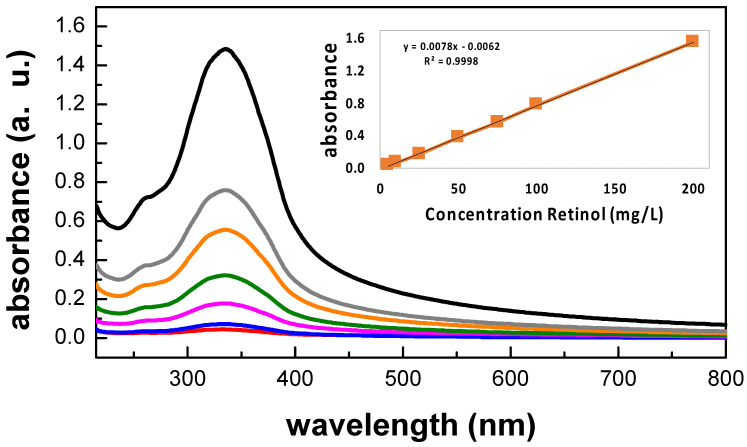
UV-Vis spectra for retinol solutions in PBS at the range of concentrations of 5 mg/L ≤ X ≤ 200 mg/L. From top to bottom, aqueous samples containing: (i)—200 mg/L (black), (ii)—100 mg/L (gray), (iii)—75 mg/L (orange), (iv)—50 mg/L (green), (v)—25 mg/L (magenta), 10 mg/L (blue), and 5 mg/L (red). The inset shows the constructed calibration curve and its curve equation from a linear regression given by Y = m X + b, with R2 > 0.99.

Figure 6 shows the typical UV-Vis spectrum of an aqueous retinol solution in PBS at 200 mg/L with the major absorption peak located at 335 nm, confirming the presence of retinol. The inset shows the chemical structure of hydrolyzed retinol which is characterized by its ***β***-ionone ring accompanied by a polyunsaturated chain consisting of four isoprenoid moieties and a terminal hydroxyl group [48,81,82]. This absorption spectrum was in total agreement with the reported literature; indeed, retinoids absorb light due to their conjugated polyene systems, with typical light absorption in regions of the ultraviolet spectrum around 325–380 nm [81,82]. Figure 7 shows the UV-Vis spectra for retinol solutions in PBS at the concentration range of 5 mg/L ≤ X ≤ 200 mg/L. The inset shows the constructed calibration curve and its curve equation from a linear regression given by *Y* = *m X* + *b*, with *R*^2^ > 0.99.

**Figure 8:**
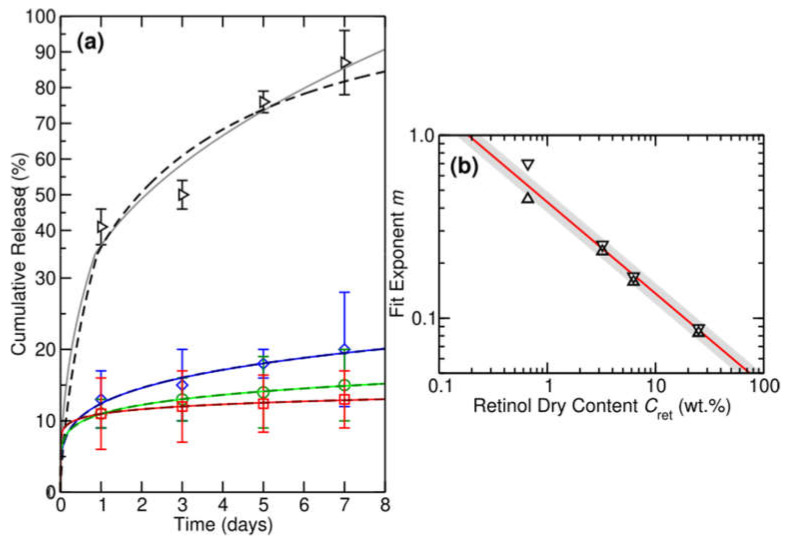
(**a**) The in vitro cumulative release percentage of retinol in PBS over 1 to 7 days for RPFM-0.1 (black triangles), RPFM-0.5 (blue diamonds), RPFM-1 (green circles), and RPFM-5 (red squares). (**b**) Shows fits of the form $1-e^{-kt^{m}}$ (dashed dark lines) and $kt^{m}+0.5kt^{2m}$ (solid light lines), with the fitted values of *m* versus retinol dry content *C*_ret_ in wt.% (triangle down and triangle up, respectively) along with a fit of the form $m\approx m_{1}/\sqrt{C_{ret}}$ (red solid line) and its standard deviation (grey region). Data are presented as standard deviations of the mean.

The abovementioned calibration curve was used to quantify the retinol released to the medium by the RPFM samples as a function of time. Figure 8A shows the in vitro cumulative release percentage of retinol as a function of the time for the set of fabricated RPFM. As observed, the fiber mats were able to release retinol in a controlled manner during the incubation period (7 days), i.e., samples exhibited a smoother burst release of retinol (a gradual increase in the cumulative release percentage). In addition, different mathematical models were used to describe the retinol release. These models provide fundamental information and play an important role in controlled release studies. In fact, they are useful in establishing the release mechanism and shed light on the development of similar controlled release systems. In Table 3 the most used mathematical models for drug delivery systems are listed [56,83,84].

In the present study, the cumulative release of retinol, *M*(*t*), should satisfy the differential equation *M*′(*t*) *= R*(*t*)*M*(*t*), where *R*(*t*) is the time-dependent rate of retinol release. The time dependence of *R*(*t*) reflects the decrease in the rate of release with time [56,83,85]. Under such general conditions, we can represent the time-dependent rate of release as a power law of the form *R*(*t*) *= kt^m−1^*, where 0 ≤ *m* ≤ 1 and *k* is the rate constant for retinol release. This yields for the cumulative release of retinol the general differential equation *M*′(*t*) *= kt^m−1^ M*(*t*) with the general solution $M\left(t\right)=Ae^{kt^{m}}+Be^{-kt^{m}}$. Applying the boundary conditions *M*(*0*) = 0 and *M*(*t*) → 1 as *t* → ∞, we obtain the general solution $M\left(t\right)=1-e^{-kt^{m}}$. For a constant release rate, i.e., *m* = 1, *M*(*t*) naturally becomes an exponential of the form $1-e^{-kt}$. It is worth noting that for *kt^m^* << 1, i.e., for *t* << *τ* where *τ* = *k*^−1/m^ is the lifetime, thus the general solution may be truncated after the first term, yielding a power law. Under these conditions *M*(*t*) ≈ *kt^m^*. For a constant rate, we find *M*(*t*) ≈ *kt*, and similarly for *m* = ½, we find *M*(*t*) ≈ *kt^1/2^*.

For its part, Figure 8B shows a direct comparison of fits of the form $1-e^{-kt^{m}}$ and $kt^{m}+0.5kt^{2m}$ with the fitted values of *m* versus retinol dry content *C*_ret_ in wt.% (triangle down and triangle up, respectively) as an inset along with a fit of the form $m\approx m_{1}/\sqrt{C_{ret}}$. These mathematical models best describe the cumulative release measured from the RPFM samples. Except for a RPFM-0.1 sample, the cumulative release remains consistently below 25% for the higher retinol loadings. This means we are in the *kt^m^* << 1 or *t* << *τ* regime, and *M*(*t*) follows a power law. In fact, from Figure 8, we see that the cumulative release generally follows a power law behavior for retinol loads above 3.23 wt.% (i.e., for samples RPFM-0.5, RPFM-1, and RPFM-5). However, for the smallest retinol loading RPFM-0.1, we reach up to an 87% cumulative retinol release. Under these conditions, *t* >> *τ*, and so *M*(*t*) should be described using the general full exponential solution. This is because a power law behavior cannot describe the high release rates, i.e., the long-time regime. Specifically, in such a case, we find that *M*(*t*) ≈ *kt^m^* predicts a full release of retinol after seven to ten days for RPFM-0.1, which is inconsistent with the physics of the problem. Table 4 provides the fitting parameters and mean absolute errors (MAE) in % for the fit of the form $1-e^{-kt^{m}}$. Overall, we find fitting the release exponent *m* yields a better description of the experimental data, as expected. We also find using the full exponential form from these fits yields a better description of the experimental results. This is most clearly the case for the smallest loading of 0.66 wt.%, where *t*~*τ*, and we are far from a power law behavior. All these findings allow us to conclude that in our case, the best mathematical models that describe the release of our system are the Fu-Kao First Order and the Peppas-Sahlin models.

It is worth noting that due to the natural degradation kinetics of retinol, the effective percentage of retinol released to the medium would be underestimated [18,45,81]. Although the samples with initial retinol content greater than 0.66 wt.% showed an apparently low release percentage, the RPFM-1 and RPFM-5 samples were able to release up to 600 μg and 1900 μg in 7 days, respectively. In the present study, the releasing pattern concluded that the release depends on time and the initial content of retinol in the samples. The present results were in total agreement with those reported in the literature. For instance, the bioactive molecules (BAMs) were efficiently released from the electrospun nanofibers via the so-called burst effect [86,87]. Moreover, in such materials, the BAM release rate could be controlled by the properties of the polymer such as hydrophilic/hydrophobic nature, biodegradability, solubility, stability, BAM distribution, micro-structure, and morphology of fibers. In addition, the mechanisms involved in the BAM release profiles include diffusion, swelling, diffusion and degradation [87]. Previously, Taepaiboon et al. investigated the release profile of retinoic acid-loaded electrospun cellulose acetate nanofiber mats [88] and demonstrated that the retinoic acid-loaded fiber mats exhibited a smoother burst release, i.e., a gradual increase in the cumulative release of retinoic acid over 6 h with the maximum retinoic acid released about 34%. It is worth noting that authors used acetate buffer solution that contained Tween 80 and methanol [88]. Similarly, Fahami et al. reported the release profile of vitamin A-loaded cress seed mucilage/PVA nanofiber in simulated gastric and intestinal fluids. According to their results, about 6% of the vitamin was released over 8 h in simulated gastric fluid while almost 16% of the vitamin was released in simulated intestinal fluid [56]. They attributed such low release trends to the high encapsulation efficiency of vitamin A in the polymer, with almost no free vitamin over the surface of the fibers, and the authors concluded that a burst release was not observed [56].

### 3.6. In Vitro Cytotoxicity Assay

**Figure 9:**
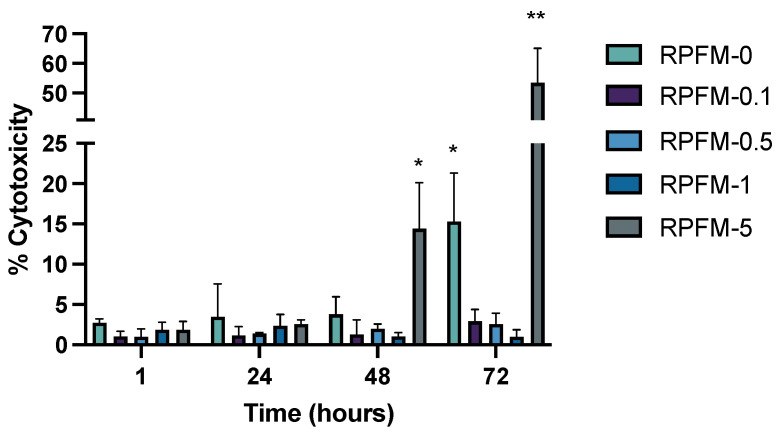
Cytotoxicity (LDH assay) assessment of *ah*-BM-MSCs cultured by indirect test with RPFM (i.e., RPFM-0, RPFM-0.1, RPFM-0.5, RPFM-1, RPFM-5) at different time periods: 1, 24, 48, and 72 h. The mean cytotoxicity percentage value was calculated and normalized with respect to the viability of cells cultured on empty plastic (TCPs) plates (positive control). Bars represent standard deviations of the mean. * Significant differences between the marked group and the other RPFM. ** Significant differences between RPFM-5 and the other RPFM at 72 h.

The viability of *ah*-BM-MSCs was evaluated using the LDH cytotoxicity assay at different time points (1, 24, 48, and 72 h) after placing the RPFM samples in indirect contact with the cells. This assay quantifies the percentage of cells that would lyse. As a matter of fact, when cells have been lysed, the LDH enzyme is released into the extracellular medium allowing its quantification by reacting with a tetrazolium salt. As shown in Figure 9, the cell viability results demonstrated that all the fabricated RPFM (except for RPFM-5 at 72 h) had no cytotoxic effects on *ah*-BM-MSCs after 72 h of treatment, i.e., the % of cytotoxicity value remained below 20% without significant differences. Indeed, it has been reported that the lower viability % value indicates a higher cytotoxic potential. Thus, the % of viability value < 70% is considered as cytotoxic to the cells [89]. It is worth noting that in spite of the fact that the control sample RPFM-0 exhibited no cytotoxic effect on cells, its viability slightly decreased for 72 h of treatment. These results are in total agreement with literature for PVA biomaterials exposed to cell culture [90]. Similarly, Gao et al. reported much higher LDH (16%) release from MC3TC-E1 cells treated with electrospun PVA scaffolds for 24 h [91]. In addition, Madruga et al. reported very similar LDH values of 8% released by ADSCs cells treated with PVA electrospun fiber mats for 24 h [92]. Surprisingly, RPFM-5 showed a cytotoxic trend effect on *ah*-BM-MSCs after 48 h, with a % of cytotoxicity value ~55% with respect to the control indicating that this sample induced partial cell lyses [93]. To support the present study, Veeramani et al. reported that the viability of 3T3 cells treated with vitamin A-rich *Pouteria camito* fruit-derived superparamagnetic nanoparticles decreased in a dose-dependent manner after 24, 48, and 72 h of cell culture [94]. Similarly, Baghirova et al. also demonstrated the dose-dependent effect of vitamin A Palmitate-loaded PLGA/Chitosan nanoparticles on HaCaT cell viability and proliferation [95]. They showed that the viability of cells treated at low doses ~10 μg·mL^−1^ for 24 h was upregulated; in contrast, cells treated at higher doses ~400 μg·mL^−1^ for 24 h exhibited a higher level of cytotoxicity, i.e., above 50% of cytotoxicity value compared to control. Thus, the present study disclosed unique information which would be valuable for future research regarding the effect of retinol-loaded PVA fiber mats with a low/high content of retinol ranging from 0 wt.% ≤ Retinol ≤ 25 wt.% on the viability of mesenchymal stem cells.

### 3.7. In Vitro Cell Proliferation

**Figure 10:**
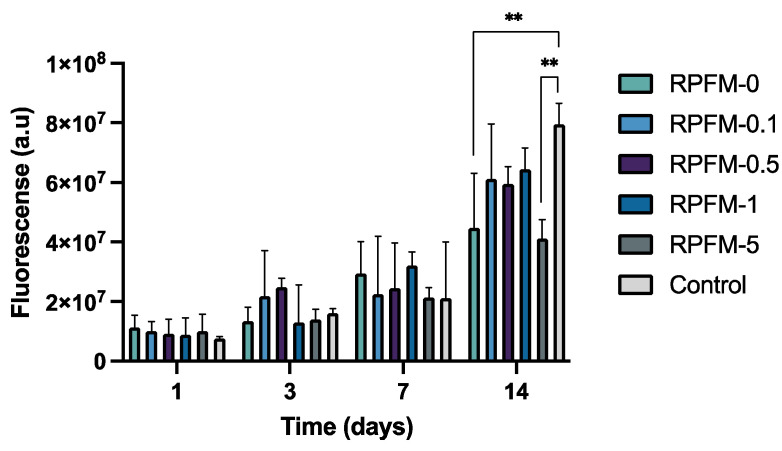
Cellular metabolic activity of *ah*-BM-MSCs cultured by indirect test with RPFM (i.e., RPFM-0, RPFM-0.1, RPFM-0.5, RPFM-1, RPFM-5) using the AlamarBlue^®^ assay at different time periods: 1, 3, 7, and 14 days. Cells cultured on empty plastic (TCPs) plates (positive control). Bars represent standard deviations of the mean. ** Significant differences between the bracketed groups at the same time period.

The cellular metabolic activity of *ah*-BM-MSCs was evaluated using a resazurin-based cell viability assay (AlamarBlue^®^) on days 1, 3, 7, and 14 after being treated with the RPFM in co-culture method. The *ah*-BM-MSCs cultured without the presence of RPFM were taken as a positive control. As observed in Figure 10, all the RPFM samples increased the cellular metabolic activity gradually (upregulated) without significant differences up to 7 days. Interestingly, however, metabolic activity was upregulated after 14 days of culture; there was a slight difference in cells treated with RPFM-0 and RPFM-5. Indeed, the metabolic activity of MSCs treated with RPFM at this specific period of time significantly decreased compared to the control, which is in agreement with the results obtained in the cytotoxicity assay. The cell proliferation and metabolic activity in the presence of biomaterials and fiber mats based on PVA have been extensively researched in the literature [91,92,96]. Previously, Scotchford et al. reported that PVA materials downregulated the proliferation rate of osteoblasts by 50% after 72 h of cell culture compared to the control [96]. Similarly, Gao et al. demonstrated that MC3TC-E1 cells proliferation treated with fibrous scaffolds of PVA was downregulated up to 75% after 72 h of cell culture compared to the control [91]. Such biological response is attributed to the purity differences of the raw material, the synthesis process, and the presence of some hydroxyl groups at the surface which can promote some oxidative stress and cell lysis [91,92,96]. On the contrary, the biological response would change once PVA is modified with bioactive molecules (BAM). Indeed, the inclusion of BAM stimulates the presence of hydrogen bonds and hydrophilic groups (for instance, carboxylic groups on the surface, similar to the proteins and molecules present in the Extracellular Matrix) [25]. Thus, PVA/BAM biomaterials would exhibit improved adhesion of cells and cellular biocompatibility [24,68,73]. These functionalities improvements are valid as long as the BAM loads stay below the threshold for cytotoxicity. Earlier studies proved the proliferative effect of MSC cells treated with PVA fiber mats [42,61,82,86,87,91,92,93]. In this context, in vitro responses of mesenchymal stem cells treated with chitosan-PVA-carbon nanotubes nanofibers were reported by Mombini et al. and found that such fiber mats exhibit suitable biocompatibility at lower loads without significant cytotoxic effects on MSCs [97,98]. Analogous results were previously reported by Shafiee et al. for MSCs treated with hybrid PVA/PCL electrospun nanofibers. Indeed, the authors investigated the cytocompatibility of scaffolds by cell adhesion and proliferation and reported that PVA/PCL scaffolds improved MSC proliferation, adhesion, and interactions after cell seeding [98].

### 3.8. Wound Healing Assays

**Figure 11:**
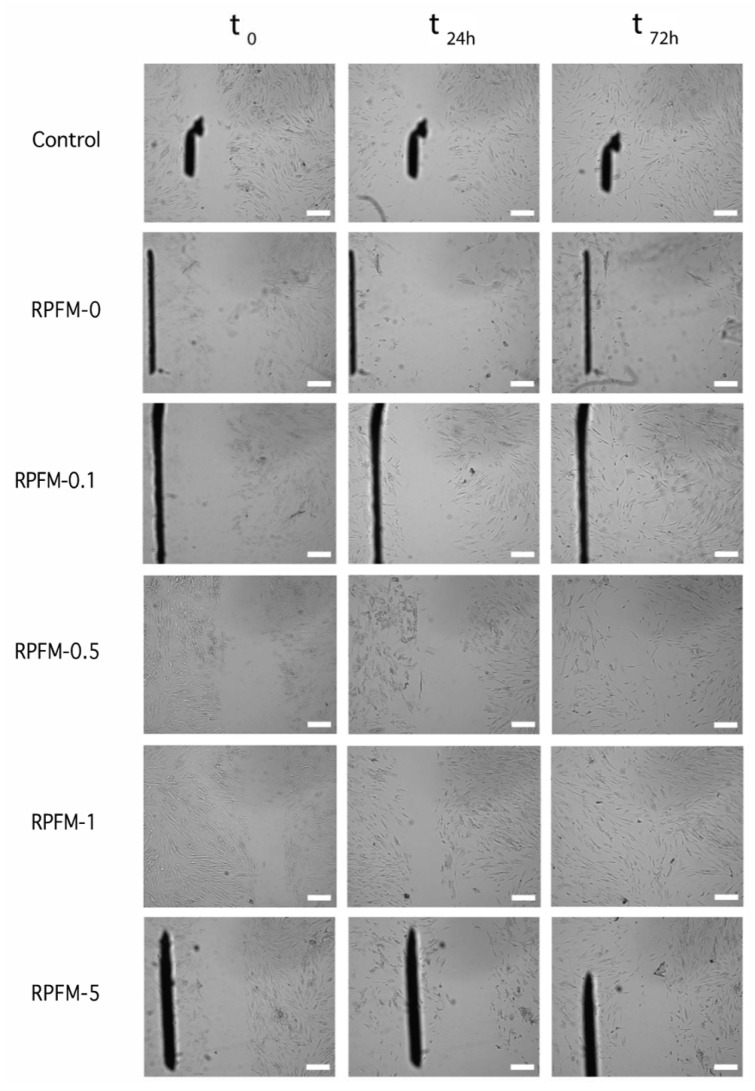
Optical microscopic phase images of *ah*-BM-MSCs migration cultured by indirect test with RPFM (i.e., RPFM-0, RPFM-0.1, RPFM-0.5, RPFM-1, RPFM-5). MSCs pre-migration (left-hand side column at t = 0) and MSCs migration after 24 h and 72 h (right-hand side columns at t = 24 h and 72 h). Cells cultured on empty plastic (TCPs) plates (positive control). Pictures correspond to the region of interest as depicted in Figure 1. Scale corresponds to 200 µm.

**Figure 12:**
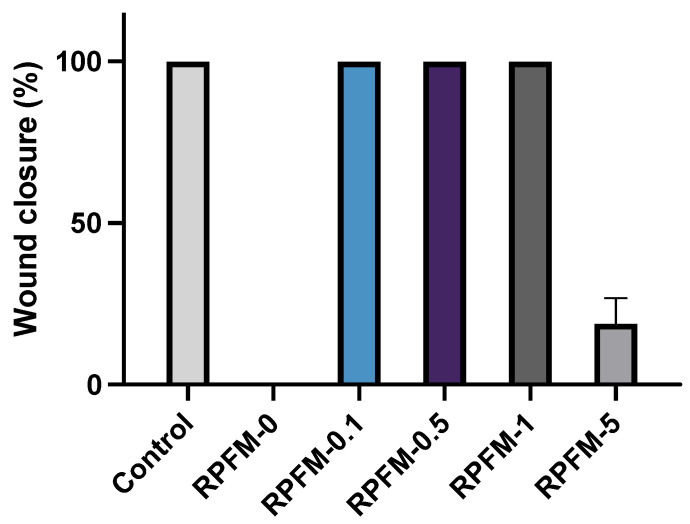
Typical plot of *ah*-BM-MSCs migration using scratch assay (as percentage of wound closure) over 72 h of exposure to RPFM samples. Data are presented as the average percent closure ± SD. Cells cultured on empty plastic (TCPs) plates (positive control). RPFM-0, RPFM-0.1, RPFM-0.5, RPFM-1, RPFM-5 represent the samples with different contents of retinol in fiber mats. RPFM-0 (material control) column is absent because MSCs treated with this material failed to heal over 72 h of culture.

Scratch assay or wound healing assay is a standard in vitro technique for cell migration assessment. In order to evaluate the potential of the prepared RPFM to enhance the migration of *ah*-BM-MSCs, a time-dependent experiment (0–72 h) was carried out. Figure 11 shows selected optical microscope phase images of MSCs pre-migration (left-hand side column at t = 0) and MSCs migration (right-hand side columns at t = 24 h and 72 h) treated with the RPFM samples loaded with different contents of retinol. The results revealed that the MSCs treated with RPFM-0.1, RPFM-0.5, and RPFM-1 exhibited similar wound healing and cell migration without significant differences compared to control (untreated cells). In contrast, the wound healing and cell migration for MSCs treated with RPFM-0 and RPFM-5 exhibited a very low cellular migration. Indeed, no wound closure was observed for MSCs after 72 h treatment with RPFM-0. To best illustrate these findings, Figure 12 shows a typical plot of *ah*-BM-MSCs migration using scratch assay (as percentage of wound closure) over 72 h of exposure to RPFM samples. The above results are in total concordance with the previous results obtained in the cytotoxicity (LDH activity) and the proliferation (AlamarBlue^®^(Thermo Fisher Scientific, Waltham, MA, USA)) assays and those reported in the literature [8,20,91,92,96,99]. Similar to the present study, earlier investigation demonstrated the wound healing effect of PVA-based fiber mats. In this context, Adeli et al. investigated the wound healing and cell migration of L-929 cells treated with electrospun PVA/chitosan/starch nanofibrous mats for 24 h [100] and demonstrated that the incorporation of starch in the fiber mats increased the cell migration, closure efficiency percentage, and healing capability [100]. Moreover, the authors also claimed that closure efficiency percentage increased in the fiber mats containing a higher ratio of chitosan. Likewise, Alavarse et al. reported the effect of wound healing capability of rabbit aortic smooth muscle cells (SMCs) treated with tetracycline hydrochloride (TCH)-loaded PVA/Chitosan electrospun fiber mats [101]. According to their finding, TCH/PVA/CS fiber mats influenced the cell migration and wound healing percentage of SMCs by retarding the complete wound closure [101]. Indeed, the rate of wound closure by TCH-loaded sample at 24 h was lowered about 10% compared to the control [101].

## 4. Conclusions

To summarize, the present study reported the synthesis, physical–chemical and biological characterization of RPFM with different contents of retinol (0 ≤ Ret ≤ 25 wt.%). The fabricated fiber mats exhibited uniform fiber diameters’ distribution within the range of 150 nm and 225 nm. In addition, the thermal stability of the fiber mats improved with increasing retinol content. In contrast, the mechanical properties such as Young’s modulus, ultimate tensile strength, and elongation at break were affected by decreasing retinol content. Indeed, for the RPFM-1 samples, the Young’s modulus was significantly reduced but the elongation at break raised up to 400%. Interestingly, all the fabricated RPFM were able to release retinol in a controlled manner and were dependent on incubation time and initial retinol content. The cell viability, proliferation, and migration results demonstrated that all the fabricated RPFM (except RPFM-5) had no cytotoxic effects and support the adhesion, proliferation, and migration of *ah*-BM-MSCs. Therefore, this study concluded that the fabricated RPFM with a retinol load in the range of 0 wt.% ≤ retinol ≤ 6.25 wt.% would be suitable candidates for skin regeneration and wound healing applications.

## Figures and Tables

**Table 1:** Summary of the weight% (wt.%) of PVA and retinol in the aqueous mixtures and their corresponding retinol-loaded PVA electrospun fiber mats (RPFM).

Sample Name	PVA in Solution (wt.%)	Retinol in Solution (wt.%)	PVA Dry Content (wt.%)	Retinol Dry Content (wt.%)
*RPFM-0*	15.00	0.00	100.00	0.00
*RPFM-0.1*	15.00	0.10	99.34	0.66
*RPFM-0.5*	15.00	0.50	96.77	3.23
*RPFM-1*	15.00	1.00	93.75	6.25
*RPFM-5*	15.00	5.00	75.00	25.00

**Table 2:** Summary of major mechanical properties as Young’s Modulus (*E*), Ultimate Tensile Strength (UTS), and elongation at break (*ε_max_*) for the retinol-loaded PVA fiber mats (RPFM) obtained from the analyses of the tensile plots.

Sample Name	E (MPa)	UTS (MPa)	*ε_max_* (%)
*RPFM-0*	44.73	2.18	79.00
*RPFM-0.1*	22.25	3.46	219.00
*RPFM-0.5*	9.39	2.84	233.00
*RPFM-1*	8.13	3.69	382.00
*RPFM-5*	9.20	2.72	186.00

**Table 3:** Mathematical models provided to describe controlled release of retinol. *M*(*t*) represents the retinol released at time *t*, *M*(∞) represents the initial amount of retinol [56,83,84].

Model Name	Mathematical Formula	Coefficients	Year
Higuchi	$\frac{M\left(t\right)}{M\left(\infty \right)}=kt^{1/2}$	k indicates the release rate constant	1963
Ritger-Peppas	$\frac{M\left(t\right)}{M\left(\infty \right)}=kt^{n}$	n represents the release (diffusion) exponent	1987
Peppas-Sahlin	$\frac{M\left(t\right)}{M\left(\infty \right)}=k_{1}t^{m}+k_{2}t^{2m}$	k_1_ and k_2_ represent diffusion and degradation rate constants and m indicates the release exponent	1989
Kopcha	$\frac{M\left(t\right)}{M\left(\infty \right)}=A\times t^{0.5}+B\times t$	A and B indicate diffusion and dissolution terms	1990
Fu-Kao Zero Order	$\frac{M\left(t\right)}{M\left(\infty \right)}=kt$	k indicates the release rate constant	2010
Fu-Kao First Order	$\frac{M\left(t\right)}{M\left(\infty \right)}=1-e^{-kt}$	k indicates the release rate constant	2010
Hixon-Crowell	$\frac{M\left(t\right)}{M\left(\infty \right)}=kt$	k indicates the release rate constant	2011

**Table 4:** Fractional retinol cumulative release power law model fits of the form 1 *− exp*(−*kt^m^*) for the fabricated RPFM samples, powers m, rate constants *k* in inverse days*^−m^*, and resulting Mean Absolute Error (MAE) in % are listed.

Sample	*m*	*k* (Days^−*m*^)	MAE (%)
RPFM-0.1	1	0.293	6.3
RPFM-0.5	1	0.039	4.3
RPFM-1	1	0.030	4.1
RPFM-5	1	0.026	4.2
RPFM-0.1	½	0.559	7.1
RPFM-0.5	½	0.092	1.7
RPFM-1	½	0.071	2.1
RPFM-5	½	0.063	2.4
RPFM-0.1	0.7	0.435	6.0
RPFM-0.5	0.252	0.133	0.5
RPFM-1	0.169	0.116	0.1
RPFM-5	0.088	0.116	0.1

## Data Availability

The data presented in this study are available on request from the corresponding author.
